# New dustywings (Neuroptera, Coniopterygidae) from mid-Cretaceous amber of Myanmar reveal spectacular diversity

**DOI:** 10.3897/zookeys.827.31961

**Published:** 2019-03-05

**Authors:** Dominika Ružičková, André Nel, Jakub Prokop

**Affiliations:** 1 Department of Zoology, Faculty of Science, Charles University, Viničná 7, 128 00 Praha 2, Czech Republic Charles University Praha Czech Republic; 2 Institut de Systématique, Évolution, Biodiversité, ISYEB – UMR 7205 – CNRS, MNHN, UPMC, EPHE, Muséum national d’Histoire naturelle, Sorbonne Universités, 57 rue Cuvier, CP 50, Entomologie, F-75005, Paris, France Sorbonne Universités Paris France

**Keywords:** Upper Cretaceous, Cenomanian, Burmite, Insecta, Neuropterida, wing venation

## Abstract

Two new genera and species of Coniopterygidae (Neuroptera) are described and illustrated from mid Cretaceous (Cenomanian) amber of Myanmar. *Mulleroconishyalina***gen. n. et sp. n.**, attributed to the Coniopteryginae, bears a unique combination of venation characters and an abdomen without plicatures. The second new genus, attributed to the Aleuropteryginae, i.e. *Palaeoconisazari***gen. n. et sp. n.**, displays a unique pattern of crossveins 1m-cua and 2mp2-cua, with the latter crossing the pigmented spot. A check-list of all fossil genera and species of Coniopterygidae is provided.

## Introduction

Neuroptera is one of few insect orders with a remarkably rich fossil history and spectacular diversity of morphotypes during the Mesozoic. The two early Permian families Permithonidae and Permoberothidae are currently considered as the stem groups of the clade Eidoneuroptera (Neuroptera + Megaloptera) (Prokop et al. 2015; Engel et al. 2018). Current views of phylogeny based on molecular and morphology datasets support the family Coniopterygidae as a sister group to Euneuroptera comprising all remaining recent families of Neuroptera (Winterton et al. 2010; Wang et al. 2017; Winterton et al. 2017, 2018; Randolf et al. 2017; Engel et al. 2018).

Coniopterygidae (dustywings) are an unusual family of Neuroptera with minute body-size, strongly reduced venation and, especially, wings covered by a waxy secretion. Dustywings are predators with larvae and adults feeding on mites, aphids, and sternorrhynchans. Currently, the family comprises 571 species assigned to 23 genera (Engel et al. 2018). They are divided into the three Recent subfamilies: Aleuropteryginae, Coniopteryginae, and Brucheiserinae, along with a single fossil one, Cretaconiopteryginae (Liu and Lu 2017). This current subdivision into four subfamilies needs to be tested by a global phylogenetic analysis of the fossil and extant taxa of this family, but it is out of the scope of the present paper. Meinander (1975) described the oldest *Juraconiopteryxzherichini* assigned to Coniopterygidae from the Upper Jurassic of Karatau in Kazakhstan, while the family is supposed to have diverged from the other Neuroptera during the Late Permian (Winterton et al. 2018). However, the record of dustywings is mainly known from amber inclusions of the following localities (see Table 1): Lower Cretaceous (Lebanon – Hammana (Neocomian), Spain – Cantabria (Albian), France – Charente – Maritime (Albian); Upper Cretaceous (Myanmar – Hukawng Valley (Cenomanian), U.S.A. – New Jersey, (Turonian), Russia – Taimyr Peninsula, (Cenomanian to Santonian), Canada – Alberta (Campanian); Eocene – India – Gujarat (Ypresian), France – Oise, Le Quesnoys (Ypresian), Baltic (Late Eocene), Ukraine – Rovno (Late Eocene); and Miocene – Dominican Republic (Burdigalian). For a long time, the Late Cretaceous amber from New Jersey was known for its high richness of neuropteran diversity (Grimaldi 2000). However, due to extensive sampling of Burmese amber during last decades it was revealed that it contains a surprisingly diverse and abundant neuropteran fauna currently attributed to 21 families, including Coniopterygidae (Grimaldi et al. 2002; Makarkin 2016; Ross 2018). Despite the fact, that the inclusions of dustywings in Burmese amber are considerably rare, six species assigned to five genera have been described so far (Ross 2018). The majority of assigned genera belong to the subfamily Aleuropteryginae. The subfamily Coniopteryginae has only two recorded genera and Brucheiserinae is still without any fossil evidence (Perrichot et al. 2014; Engel 2004, 2016; Makarkin 2016; Liu and Lu 2017, see Table 1).

**Table 1.** List of extinct genera and species of Coniopterygidae.

MESOZOIC	
JURASSIC	
Subfamily Aleuropteryginae	
*Juraconiopteryx* Meinander, 1975	Callovian/Oxfordian; Kazakhstan
†*J.zherichini* Meinander, 1975	
CRETACEOUS	
Subfamily Aleuropteryginae	
*Achlyoconis* Engel, 2016	Cenomanian; Myanmar
†*A.heptatrichia* Engel, 2016	
*Alboconis* Nel et al., 2005	Albian; France
†*A.cretacica* Nel et al., 2005	
*Apoglaesoconis* Grimaldi, 2000	Turonian; U.S.A.
†*A.ackermani* Grimaldi, 2000	
†*A.cherylae* Engel, 2002	Turonian; U.S.A.
†*A.luzzii* Grimaldi, 2000	Turonian; U.S.A.
†*A.swolenskyi* Grimaldi, 2000	Turonian; U.S.A.
*Garnaconis* Perrichot & Nel in Perrichot et al. 2014	Turonian; France
†*G.dupeorum* Perrichot & Nel, 2014	
*Glaesoconis* Meinander, 1975	Cenomanian; Myanmar
†*G.baliopteryx* Engel, 2004	
†*G.cretica* Meinander, 1975	Santonian; Russia
†*G.nearctica* Grimaldi, 2000	Turonian; U.S.A
†*G.popovi* Makarkin & Perkovsky, 2017	Santonian; Russia
*Libanoconis* Engel, 2002	Barremian; Lebanon
†*L.fadiacra* Whalley, 1980	
†*L.siberica* Makarkin & Perkovsky, 2019	Cenomanian; Russia
*Palaeoconis* Ružičková, Nel & Prokop, n. gen.	Cenomanian, Myanmar
†*P.azari* Ružičková, Nel & Prokop, n. sp.	
Subfamily Coniopteryginae	
*Jurasiatypus* Kaddumi, 2005	Albian; Jordan
†*J.cretatus* Kaddumi, 2005	
*Libanosemidalis* Azar et al. 2000	Barremian; Lebanon
†*L.hammanaensis* Azar et al. 2000	
*Mulleroconis* Ružičková, Nel & Prokop, n. gen.	Cenomanian, Myanmar
†*M.hyalina* Ružičková, Nel & Prokop, n. sp.	
*Paranimboa* Engel, 2016	Cenomanian; Myanmar
†*P.litotes* Engel, 2016	
†*P.groehni* Sziráki, 2016	Cenomanian; Myanmar
*Phtanoconis* Engel, 2004	Cenomanian; Myanmar
†*P.burmitica* Engel, 2004	
Subfamily Cretaconiopteryginae	
*Cretaconiopteryx* Liu & Lu, 2017	Cenomanian; Myanmar
†*C.grandis* Liu & Lu, 2017	
CENOZOIC	
Subfamily Aleuropteryginae	
*Archiconiocompsa* Enderlein, 1910	Priabonian; Russia
†*A.prisca* Enderlein, 1910	
*Archiconis* Enderlein, 1930	Priabonian; Russia
†*A.electrica* Enderlein, 1930	
*Geroconiocompsa* Engel, 2010	Priabonian; Russia
†*G.ostara* Engel, 2010	
*Hemisemidalis* Meinander, 1972	Priabonian; Poland
†*H.kulickae* Dobosz & Krzemiński, 2000	
*Neoconis* Enderlein, 1930	Burdigalian/Langhian; Dominican Republic
†*N.paleocaribis* Grimaldi & Engel, 2013	
*Pararchiconis* Nel, 1991	Rupelian; France
†*P.quievreuxi* Nel, 1991	
*Spiloconis* Enderlein, 1907	Burdigalian/Langhian; Dominican Republic
†*S.glaesaria* Meinander, 1998	
†*S.oediloma* Engel & Grimaldi, 2007	Burdigalian/Langhian; Dominican Republic
†*S.eominuta* Grimaldi & Engel, 2013	Ypresian; India
Subfamily Coniopteryginae	
*Coniopteryx* Curtis, 1834	Burdigalian/Langhian; Dominican Republic
†*C.antiquua* Engel & Grimaldi, 2007	
†*C.timidus* Hagen, 1856	Priabonian; Poland
*Neosemidalis* Enderlein, 1930	Holocene, Benin
†*N.enderleini* Meunier, 1910	
*Parasemidalis* Enderlein, 1905	Ypresian; France
†*P.eocenica* Nel et al., 2005	
†*P.sharovi* Meinander, 1975	Priabonian; Russia
*Semidalis* Enderlein, 1905	Priabonian; Russia
†*S.fritschi* Enderlein, 1930	

Herein we report two new genera and species of Aleuropteryginae and Coniopteryginae from the Cenomanian amber of Myanmar. These new taxa are based on morphological characters with special attention to the wing venation and structure of the antennae.

## Material and methods

All herein examined specimens are preserved in Burmese amber recovered from the deposits in northern Myanmar (Hukawng Valley, Kachin) (Cruickshank and Ko 2003; Grimaldi and Ross 2017). The age of these fossiliferous layers was previously considered as Late Albian or Early Cenomanian on the basis of palynomorphs (Cruickshank and Ko 2003), and recently confirmed as the lowermost Cenomanian (98.79 ± 0.62 Ma) by radiometric analysis of zircons (Shi et al. 2012). The record from this locality has recently been reviewed by Grimaldi and Ross (2017). Contemporary investigations on various insect lineages has emphasized the tremendous diversity and disparity of the entomofauna (e.g., Grimaldi and Ross 2017), as well as its impact on an understanding of changes in Late Cretaceous biotas along with the Late Cretaceous-Paleogene faunal turnover, such as replacement by specialized angiosperm pollinators and evidence of remarkable parasitoid strategies (e.g., Grimaldi et al. 2002; Batelka et al. 2016, 2018, in press; Huang et al. 2016; Makarkin 2016).

The material was studied with Leica MZ12.5 stereomicroscope and Olympus BX40 microscope, and photographed using a Canon D550 digital camera mounted on a tripod and coupled with a MP-E 65 mm macro-lens, or attached to an Olympus BX40. The original photographs were processed using Adobe Photoshop CS4, while some images we prepared a series of focal layers which were them combined using the focus-stacking software packages Helicon Focus Pro or Zerene Stacker. The specimens reported herein are originally from the private collection of Patrick Müller, Käshofen, Germany (accession numbers abbreviated as BUB – Burmese Bernstein). All type specimens are deposited in the Museum für Naturkunde, Berlin.

Terminology of wing venation nomenclature and interpretation of veins follow Breitkreuz et al. (2017) who studied the wing venation and tracheation across the neuropteran families. Abbreviations of longitudinal veins: C – costa, ScP – subcosta posterior, R – radius, RA/ RP – radius anterior/ posterior, M – media, MA/ MP – media anterior/ posterior, Cu – cubitus, CuA/CuP – cubitus anterior/ posterior, A – anal vein.

## Systematics

### Order Neuroptera Linnaeus, 1758

#### Family Coniopterygidae Burmeister, 1839

##### Subfamily Coniopteryginae Burmeister, 1839

###### Mulleroconis gen. n.

Taxon classificationAnimaliaNeuropteraConiopterygidae

http://zoobank.org/CC3D1487-6C5F-4998-BDF4-F52D6E8731BE

####### Type species.

*Mulleroconishyalina* gen. et. sp. n.

####### Diagnosis.

Forewing hyaline; one straight crossvein in proximal part of costal area; ScP2 diverges obliquely from ScP1; crossvein ra-rp absent; crossvein rp-ma undulated; M without macrosetae reaching posterior wing margin with two branches; crossvein cua-cup straight and aligned with 2cup-a1.

####### Etymology.

The generic name is a combination collector’s surname (Müller) and the Greek ‘conis’ meaning dust. The generic name is feminine in gender.

###### Mulleroconis hyalina

Taxon classificationAnimaliaNeuropteraConiopterygidae

gen. et. sp. n.

http://zoobank.org/F8CCB036-E564-4C4A-94E2-3C50CD92C8E6

Figures 1 , 2

####### Holotype.

BUB 2907; lowermost Cenomanian amber (Shi et al. 2012); Myanmar, Kachin, Hukawng Valley; preserved in a polished, transparent yellow piece of amber (5.68 × 5.00 × 1.09 mm), deposited in the Museum für Naturkunde, Berlin (ex. coll. Patrick Müller). The holotype is in amber syninclusion with one representative of the Auchenorrhyncha.

####### Etymology.

The specific epithet is after the hyaline forewing membrane.

####### Diagnosis.

As for the genus (*vide supra*).

####### Description.

Male. Body length ca. 1.17 mm (measured from tip of head to tip of genitalia). Head poorly preserved, only the last three segments of one maxillary palp are visible and the terminal segment is distinctly broader and longer than two remaining. Length of terminal palpomere ca. 0.09 mm. Thorax length ca. 0.28 mm.

Forewing ca. 1.46 mm long, ca. 0.65 mm wide; ScP1 long and parallel to costal margin; Scp2 present; division of R into RA and RP at about one-third wing length; RA simple distally connected to ScP2, parallel to ScP1; RP forked; rp-ma markedly sinuate, near the fork RP1 and RP2, connected to MA; stem of M running close to stem of R, briefly connecting each other, M distally branched into MA and MP, without stiff setae; crossveins 1m–cua and 2m-cua present, 1m–cua near the base of wing, between M and CuA, 2m-cua slightly sinuate, between M and CuA, situated slightly behind midwing; CuP separated from CuA near the base of wing; cua-cup present, located at level of division veins RA and RP; crossveins 1cup-a1 and 2cup-a1 present, 1cup-a1 connected to CuP near to fork of CuA and CuP, 2cup-a1 almost aligned with cua-cup; A1 and A2 clearly connected near the base of wing, a1-a2 present. Hindwing ca. 1.25 mm long, ca. 0.55 mm wide with venation very similar to forewing, differing in position of sinuate crossvein rp2-ma; crossvein m-cua partially preserved and basal part of wing with hardly recognizable venation pattern. Legs slender; fore femora (only left femur is visible) a bit shorter and wider than femora of second and third pair of legs; tibias covered with setae; tarsi five-segmented; first tarsomere distinctly longer than remaining tarsomeres; fifth tarsomere elongated with two apical claws. Abdomen large, length 0.64 mm, width 0.22 mm, including genitalia, with widest part approximately in middle of its length, greatly tapering to narrow apical segments; abdominal plicatures absent. Genital structures hardly discernible, presumably below projection of gonarcus, ultimate apices of parameres visible from dorsal view.

**Figure 1.**
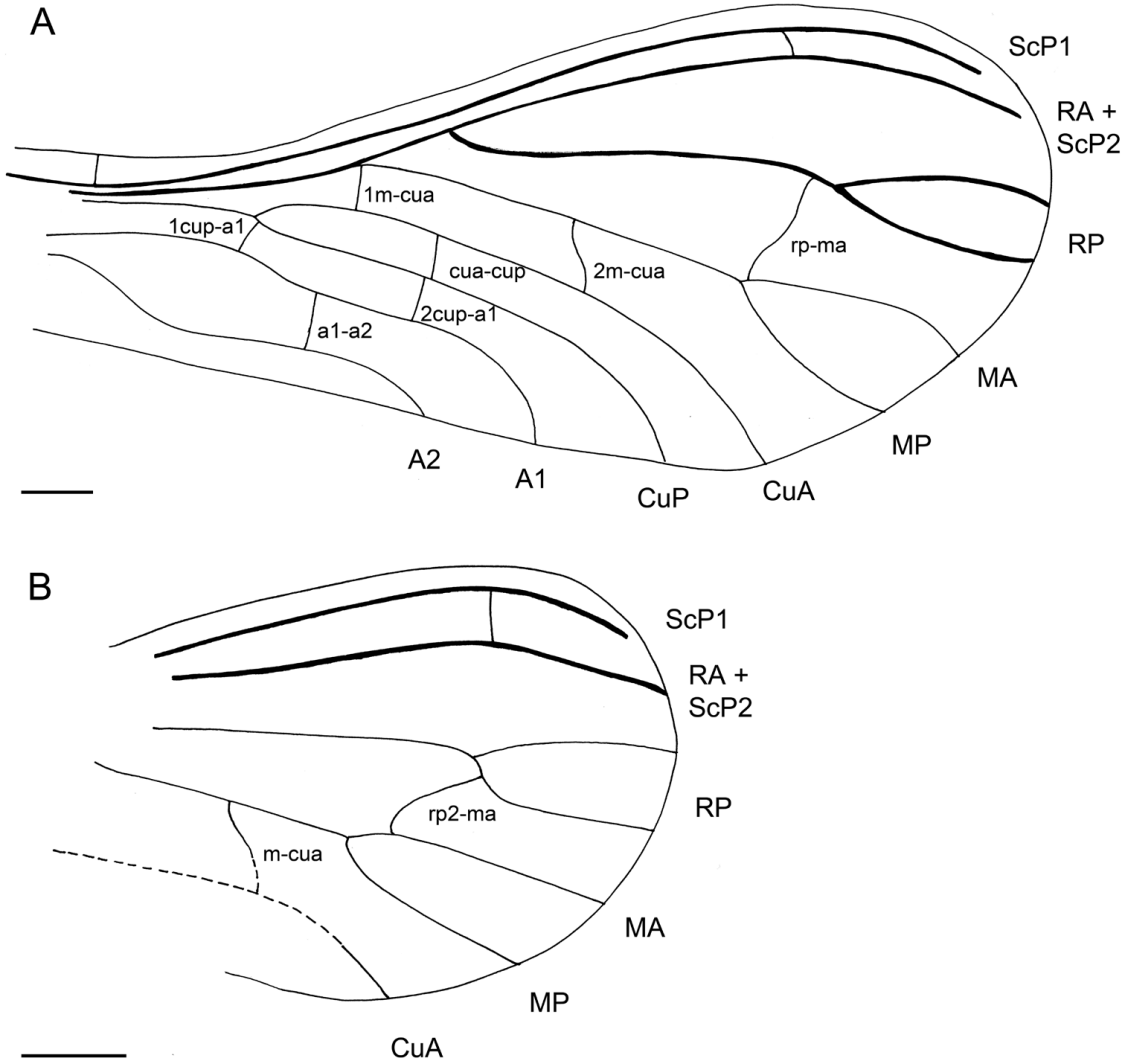
*Mulleroconishyalina* gen. et. sp. n., holotype BUB 2907. **A** Drawing of left fore wing **B** drawing of left hindwing. Scale bar: 100 µm.

**Figure 2.**
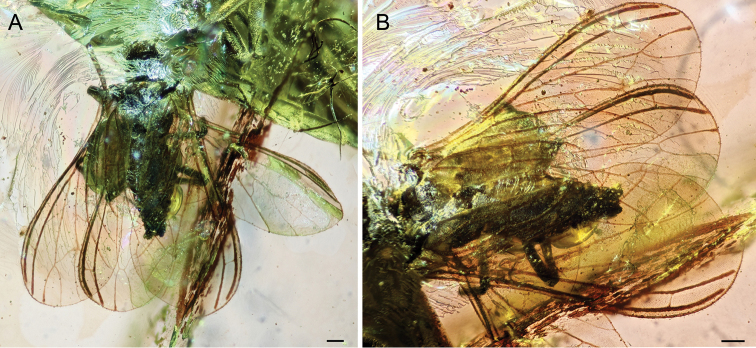
*Mulleroconishyalina* gen. et. sp. n. holotype BUB 2907. **A** Habitus dorsally **B** wing venation – detail. Scale bar: 100 µm.

####### Discussion.

*Mulleroconis* gen. n. can be attributed to the Coniopteryginae based on the following combination of forewing characters: M is bifurcate (a trait occurring in almost all members of this subfamily) (Makarkin and Perkovsky 2019), the absence of two macrosetae on the media, the presence of only one crossvein between RP and M (in our species between RP and MA), and the absence of the abdominal plicatures. The Mesozoic record of Coniopteryginae currently comprises four genera, i.e. *Jurasiatypus* Kaddumi, 2005; *Libanosemidalis* Azar et al. 2000; *Paranimboa* Engel, 2016 and *Phthanoconis* Engel, 2004 (see list below). All four genera share the presence of two basal crossveins in the costal space, unlike *Mulleroconis* gen. n. In addition, *Libanosemidalis*, *Paranimboa* and *Jurasiatypus* differ from *Mulleroconis* gen. n. in the presence of one crossvein between RA (ScP2) and RP. *Libanosemidalis*, described from the Lower Cretaceous amber of Lebanon, differs from *Mulleroconis* gen. n. in the presence of rp2-ma while in *Mulleroconis* gen. n. the crossvein rp-ma is shifted proximally, followed by a single crossvein between M and CuA and the presence of crossvein a2 connecting A2 and the hind margin of the wing (Azar et al. 2000). *Paranimboa* and *Phthanoconis* are both known from the Cenomanian amber of Myanmar (Engel 2016; Sziráki 2016, 2017). *Paranimboa* has a simple RP unlike in *Mulleroconis* gen. n. where the RP has two terminal branches. Furthermore, it differs in the position of the crossvein between RP and M and in the position of crossvein 1cup-a1 which is branching off from point where Cu is divided into CuA and CuP and in the presence of crossvein a2 (Engel 2016; Sziráki 2016, 2017).

*Phthanoconis* and *Jurasiatypus* both differ from *Mulleroconis* gen. n. in the presence of a single crossvein between M and CuA and in the complete absence of crossveins between CuA and CuP, CuP and A1, as well as between A1 and A2. Moreover, *Phthanoconis* differs from *Mulleroconis* gen. n. in the absence of a crossvein between RP and M (Engel 2004). *Jurasiatypus* was based on single found specimen (holotype) from the Lower Cretaceous amber of the Kurnub in Jordan (Kaddumi 2005). It differs from *Mulleroconis* gen. n. in the position of the crossvein between RP2 and MA (Kaddumi 2005).

##### Subfamily Aleuropteryginae Enderlein, 1905

###### Palaeoconis gen. n.

Taxon classificationAnimaliaNeuropteraConiopterygidae

http://zoobank.org/D2E45555-5EB3-4C36-A9D9-D448DD3D6675

####### Type species.

*Palaeoconisazari* gen. et. sp. n.

####### Diagnosis.

Antennae with 19 flagellomeres. Forewing with three pigmented spots; two crossveins present in apical part of costal area. Crossvein rp2-ma approximately as long as basal abscissa of RP2. Media with one macroseta, ending with three terminal branches. Crossveins 1m-cua, 2mp2-cua and a1 present. Abdomen with discernible plicatures on sternites II-IV.

####### Etymology.

The generic name is a combination of Palaeo and the suffix conis meaning dust.

###### Palaeoconis azari

Taxon classificationAnimaliaNeuropteraConiopterygidae

gen. et. sp. n.

http://zoobank.org/D2FC29B5-FFBE-42C7-AD0B-FD637B5011E7

Figures 3 , 4

####### Holotype.

BUB 2914; lowermost Cenomanian amber (Shi et al. 2012); Myanmar, Kachin, Hukawng Valley; preserved in a polished, transparent yellow piece of amber (9.18 × 6.77 × 2.18 mm), deposited in the Museum für Naturkunde, Berlin (ex. coll. Patrick Müller). The amber piece contains also one syninclusion of an imago of Diptera.

####### Etymology.

The specific epithet honors Prof. Dany Azar (Lebanese University, Fanar, Lebanon), friend and colleague of AN and JP and worldwide known palaeoentomologist.

####### Diagnosis.

As for the genus (*vide supra*).

####### Description.

Male. Body length ca. 1.93 mm (measured from tip of head to tip of genitalia). Head hypognathous, ca. 0.34 mm. Compound eyes well developed, 0.22 mm x 0.14 mm (from lateral view). Antennae 21-segmented (with 19 flagellomeres), scape and pedicel stouter, longer and broader than flagellomeres, first flagellomere longer and wider than remaining flagellomeres, flagellomeres subquadrate, nearly as long as wide, terminal flagellomere conical. Maxillary palps five-segmented, fifth segment distinctly larger than other palpomeres, length of fifth segment ca. 0.12 mm. Labial palps three-segmented, third segment larger than remaining. Thorax well developed. Prothorax narrower and overall smaller than meso- and metathorax.

**Figure 3.**
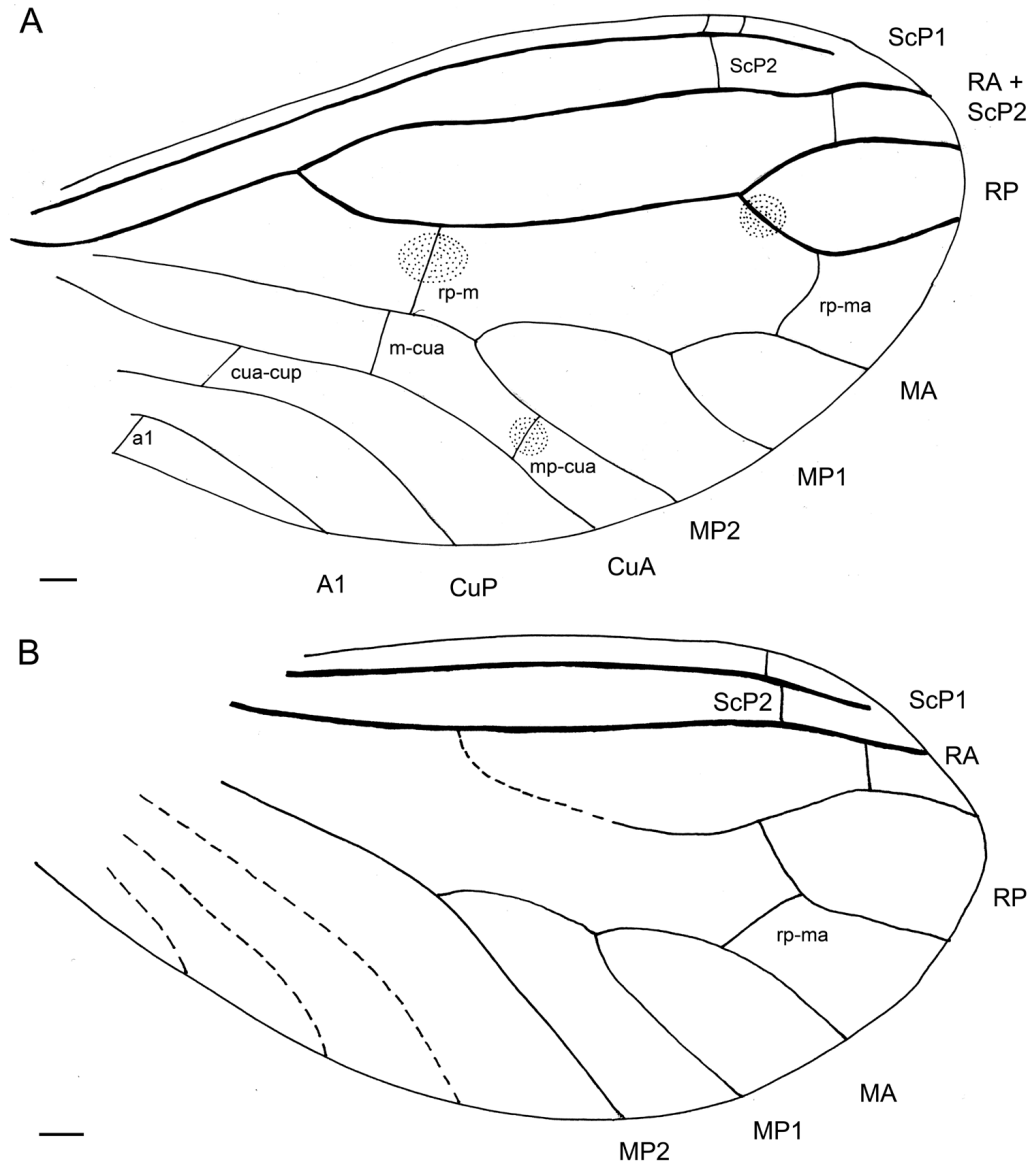
*Palaeoconisazari* gen. et sp. n., holotype BUB 2914. **A** Drawing of right fore wing **B** drawing of left hindwing. Scale bars: 100 µm.

**Figure 4.**
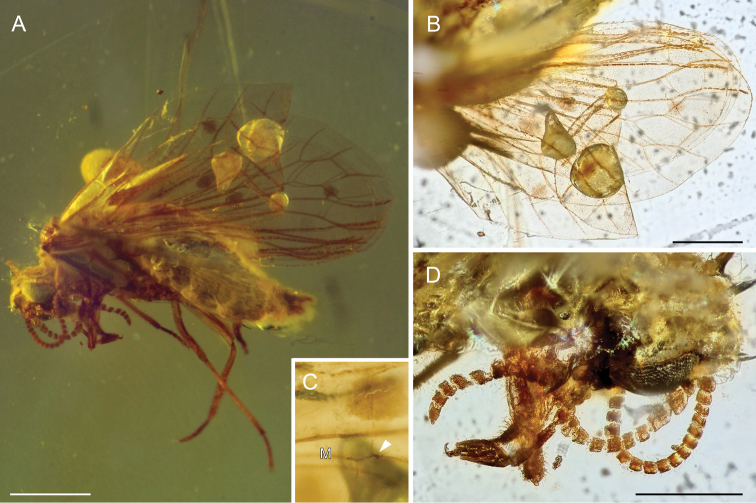
*Palaeoconisazari* gen. et sp. n., holotype BUB 2914. **A** Habitus lateral view **B** wing venation and spots **C** detail of forewing venation with stiff seta on vein M **D** head from dorso-lateral view showing antennae, terminal segment of maxillary palp and eye. Scale bars: 500µm (**A, B**); not in scale: (**C**); 50 µm (**D**).

Forewing ca. 2.19 mm long, ca. 0.93 mm wide; two distinct apical crossveins in costal area; division of ScP1 and ScP2 0.31 mm from wing apex; R branching into RA and RP 0.48 mm from wing base; RA simple, distally ending connecting with branch of ScP2; RP branched into RP1 and RP2; ra-rp1 present; first spot on basal abscissa of RP2 near RP1 (near place where is RP forked); crossvein rp-m oblique crossing dark spot about mid-length; M with three branches (MA, MP1 and MP2), M with one stiff seta near crossvein rp-m (Figs 3A, 4C); crossvein rp2-ma relatively long, approximately as long as basal abscissa of RP2; crossvein 1m–cua near rp-m, but not aligned; crossvein 2mp2-cua near to wing apex, with third spot over it; crossveins cua-cup and a1 present. Hindwing ca. 2.12 mm long, ca. 0.92 mm wide; venation pattern very similar to forewing with exception of only single crossvein between C and ScP, wing membrane in hindwing hyaline without any pigmented spots; crossveins ra-rp1 and rp2-ma present; basal part of hindwing without clearly recognizable venation.

Legs long and slender; femora covered with fine and sparse setae; tibiae covered with stiff and dense setae; tarsi five-segmented, first tarsomere distinctly longer than remaining tarsomeres, terminal tarsomere elongated ending with two claws, tarsi covered with fine dense setae. Abdomen large and broad, tapered to the end (visible from lateral view only); plicatures present on sternites II-IV; length of abdomen including genitalia 1.22 mm, width 0.54 mm. Structures of external genitalia hardly discernible with exception of domed and well-sclerotized ectoproct in lateral view and the caudal projection of gonarcus below.

####### Discussion.

*Palaeoconis* gen. n. can be attributed to the Aleuropteryginae based on the presence of two crossveins between RP and M and also the presence of plicatures on abdominal sternites II-IV. Nevertheless, Zimmermann et al. (2009) considered this last character as a symplesiomorphy of the Coniopterygidae. However, *Palaeoconis* does not possess in the forewings two long stiff setae on M, which is the diagnostic character for Aleuropteryginae. In contrast to this, the absence of these setae on the media and the presence of three-branched media are considered as plesiomorphic characters typical for Mesozoic Coniopterygidae (Engel 2002). In addition, *Palaeoconis* possesses in forewing crossvein a1 connecting A1 to posterior wing margin. According to Engel (2016) the subfamily Aleuropteryginae contains 14 genera (Mesozoic and Cenozoic), i.e., *Achlyoconis* Engel, 2016; *Alboconis* Nel et al., 2005; *Apoglaesoconis* Grimaldi, 2000; *Archiconiocompsa* Enderlein, 1910; *Archiconis* Enderlein, 1930; *Garnaconis* Perrichot and Nel in Perrichot et al., 2014; *Geroconiocompsa* Engel, 2010; *Glaesoconis* Meinander, 1975; *Hemisemidalis* Meinander, 1972; *Juraconiopteryx* Meinander, 1975; *Libanoconis* Engel, 2002; *Neoconis* Enderlein, 1930; *Pararchiconis* Nel, 1991, and *Spiloconis* Enderlein, 1907.

*Achlyoconis*, *Alboconis*, *Apoglaesoconis*, *Glaesoconis*, and *Libanoconis* share the presence of two distinct basal crossveins in the costal area, and in addition *Achlyoconis, Alboconis* and *Libanoconis* share the presence of crossvein a1–a2, vein A2 and crossvein a2. These traits separate these genera from *Palaeoconis* gen. n. In addition, *Glaesoconis* described from the Lower Cretaceous Myanmar amber differs from *Palaeoconis* gen. n. by a distinctly shorter crossvein rp2-ma , single crossvein m–cua, presence of A2 and four pigmented spots on wing membrane instead of three in *Palaeoconis* (Engel 2004). *Libanoconis*, known from the Lower Cretaceous amber of Lebanon, differs from *Palaeoconis* gen. n. in the positions of crossveins between M and CuA (1m–cua is near to wing base; 2m–cua approximately at mid-length of wing), and lack pigmented spots on membrane (Engel 2002; Nel et al. 2004). *Achlyoconis* described from the Upper Cretaceous amber of northern Myanmar differs from *Palaeoconis* gen. n. in the presence of seven thickenings with specialized setae on M, positions of crossveins 1m-cua and 2m-cua, presence of a single crossvein between CuP and A1, and four pigmented spots (Engel 2016). *Alboconis*, described from the Lower Cretaceous French amber, differs from *Palaeoconis* gen. n. by a two branched media bearing two long setae, crossvein between CuA and R+M, and two crossveins between CuP and A1 (Nel et al. 2004). *Apoglaesoconis* and *Garnaconis* share the following features such as M with two long setae, presence of crossveins cup–a1; a1–a2, vein A2 and absence of pigmented spots on wing membrane. *Apoglaesoconis*, from the Late Cretaceous amber of New Jersey, differs from *Palaeoconis* gen. n. by one crossvein between RP and M, branches RP2 and MA forming a cross, and by a single crossvein between M and CuA (Engel 2002). *Garnaconis*, from the Late Cretaceous amber of Vendée in northwestern France, differs from *Palaeoconis* gen. n. in the remote positions of separated transverse ScP2 and crossvein between RA+ScP2 and RP1 (see Fig. 3), unlike both being aligned in *Garnaconis*. *Palaeoconis* gen. n. bears two crossveins beween RP and M, a two-branched media with setae instead of only one crossvein between RP2 and MA and a three-branched media without setae in *Garnaconis* (Perrichot et al. 2014). *Juraconiopteryx*, Upper Jurassic of Kazakhstan, represents the oldest record of Coniopterygidae. Wing venation of *Juraconiopteryx* is very poorly preserved (Meinander 1975), therefore we are unable to make a reliable comparison with *Palaeoconis*.

## Conclusion

In this contribution we extended our knowledge on past diversity of Coniopterygidae. Two new genera and species assigned to the two subfamilies Aleuropteryginae and Coniopteryginae are herein described and illustrated from the Early Cenomanian amber of Myanmar. These new taxa are established mainly on the basis of wing venation patterns, number of antennal flagellomeres along with other body structures like abdominal plicatures. Our research uncovers spectacular diversity of Coniopterygidae in mid-Cretaceous ecosystems and in the same time supports the antiquity of this group that seems to have had a remarkable evolutionary stasis since the mid-Cretaceous.

## Supplementary Material

XML Treatment for Mulleroconis

XML Treatment for Mulleroconis hyalina

XML Treatment for Palaeoconis

XML Treatment for Palaeoconis azari
